# Suppression of cardiocirculatory responses to orthostatic stress by passive walking-like leg movement in healthy young men

**DOI:** 10.1186/1880-6805-31-24

**Published:** 2012-09-12

**Authors:** Hisayoshi Ogata, Ikuyo Fujimaru, Keiko Yamada, Takaharu Kondo

**Affiliations:** 1 Department of Lifelong Sports for Health, College of Life and Health Sciences, Chubu University, 1200 Matsumoto-cho, Kasugai-shi, Aichi, 487-8501, Japan

**Keywords:** Standing posture, Orthostatic intolerance, Passive exercise, Rehabilitation

## Abstract

**Background:**

Although passive walking-like leg movement in the standing posture (PWM) has been used in the clinical field, the safety of PWM has not been fully determined despite the risks of orthostatic intolerance due to standing posture. The aim of the present study was to examine cardiocirculatory response during PWM in healthy young men.

**Methods:**

The subjects (n = 13) spent 5 min in a sitting position and then 5 min in a quiet standing position to determine baseline levels. Thereafter, they underwent 25-min rhythmic PWM at 1 Hz while standing. In another bout, subjects experienced the same protocol except that they underwent 25-min quiet standing (QS) instead of 25-min PWM. Two subjects dropped out of the 25-min QS due to feeling of discomfort. Thus, data obtained in the remaining eleven subjects are presented.

**Results:**

In the PWM trial, systolic arterial blood pressure (SAP) decreased from 112 ± 8 mmHg during the sitting baseline period to 107 ± 8 mmHg during the standing baseline period (p <0.05), while heart rate (HR) increased from 73 ± 9 bpm during the sitting baseline period to 84 ± 10 bpm during the standing baseline period (p <0.001). After the imposition of PWM, SAP increased from 107 ± 8 mmHg in the standing baseline period to 120 ± 6 mmHg (p <0.001), while HR decreased from 84 ± 10 bpm in the standing baseline period to 76 ± 9 bpm (p <0.05). In the QS trial, SAP, which had decreased during the standing baseline period compared to that during the sitting baseline period, remained lowered during the 25-min QS period, while HR, which had increased during the standing baseline period compared to that during the sitting baseline period, remained elevated during the 25-min QS period. In both bouts, HR showed almost mirror-image changes in the high-frequency component of HR variability, suggesting that the changes in HR were due to change in parasympathetic activation. Double product (HR × SAP), as a predictor of myocardial oxygen consumption, during the 25-min QS period tended to increase with time, but double product remained almost constant during the 25-min PWM period.

**Conclusions:**

The results suggest that PWM is effective for suppressing cardiocirculatory responses to orthostatic stress.

## Background

Passive walking-like leg movement in the standing posture (PWM) has been thought to have the potential to prevent disuse syndrome including muscle atrophy, bone mass loss, joint contracture and pressure sores [1]. On the other hand, the safety of PWM has not been fully determined despite the risks of orthostatic intolerance due to standing posture. Since cardiocirculatory response to orthostatic stress may eventually trigger orthostatic intolerance [2], it is important to examine cardiocirculatory responses to PWM.

Quiet standing posture induces a pooling of blood in the lower extremities, resulting in decreases in central blood volume (volume of blood in the pulmonary vessels and the four chambers of the heart), stroke volume, cardiac output and systolic arterial blood pressure [3]. Heart rate (HR) increases to maintain cardiac output [3]. Against these responses, it was shown that PWM induces pressor response and that HR, which is increased by postural change from sitting to standing, is reduced by PWM [1]. Thus, we hypothesized that cardiocirculatory responses to standing posture, which are pronounced during quiet standing, are suppressed by PWM. In this study, in order to test this hypothesis, we examined changes in HR variability as a measure of cardiac autonomic nerve activity [4] and double product (DP) (HR × systolic arterial blood pressure) as a predictor of myocardial oxygen consumption in normal young men during upright exercise under a wide variety of circumstances [5] in addition to HR and blood pressure. We paid special attention to young people as subjects. The reason for this is as follows. One cause of orthostatic intolerance during standing posture is vasovagal response. The vasovagal response is development of arteriolar dilation and inappropriate cardiac slowing leading to arterial hypotension with loss of consciousness [6]. The frequency of vasovagal response during tilting has been found to be higher in young people than in the elderly people [7]. In addition, epidemiological evidence suggests a higher incidence of reflex syncope, including vasovagal syncope, carotid sinus syncope and situational syncope, in teenagers and adolescents than in the elderly [8]. Thus, there is a greater need for examination of cardiocirculatory responses to orthostatic stress in young people for prevention of vasovagal response.

## Methods

### Subjects

Thirteen healthy young male subjects with a mean age ± standard deviation (SD) of 18.8 ± 0.8 years, a mean weight of 62.5 ± 6.2 kg, and a mean height of 169.7 ± 4.2 cm participated in this study. All of the subjects were nonsmokers. Voluntary consent for participation in this study was obtained from all subjects after they were informed of the purpose of the experiment, the procedure and possible risks. The study was conducted in accordance with the Helsinki Declaration and was approved by the Ethics Committee of Chubu University in Kasugai-shi, Aichi, Japan.

### Experimental protocols

The subjects refrained from eating for at least 3 hours before the test, taking caffeine for at least 5 hours before the test, and drinking alcohol or doing heavy exercise for 12 hours before the test. The temperature in the experimental room was set to 21 to 25°C.

**Figure 1:**
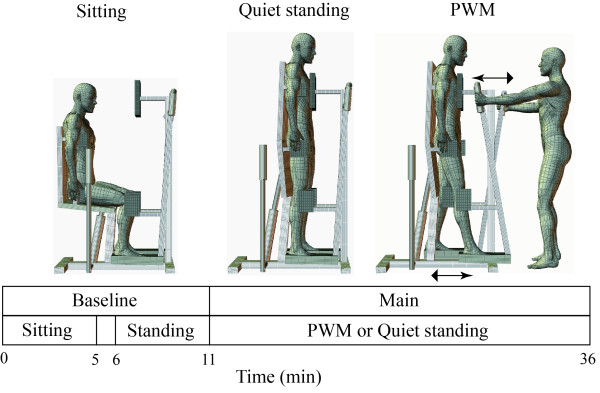
**Experimental protocol.** Subjects spent 5 minutes in a sitting position and this was followed by a 5-minute quiet standing period with a 1-minute transition period for postural change between sitting and standing. Thereafter, the subjects underwent 25-minute rhythmic PWM or 25-minute quiet standing. A commercially available device was used for PWM and quiet standing. The device enables subjects to change their posture from sitting to standing by pulling a built-in hydraulic lever. Standing posture is stabilized by fixing the trunk, pelvis and knees using front, lateral and back trunk pads, lateral pelvic pads, and kneepads. Bilateral handles located in front of the trunk are linked to the footplates, thus allowing one leg to move forward while the other moves back by pushing and pulling the handles alternately. PWM: passive walking-like leg movement.

Quiet standing (QS) and PWM were carried out using a commercially available device (Easy Stand Glider, Altimate Medical, Inc., Morton, MN, USA) as shown in Figure 1. Briefly, this device enables subjects to change their posture from sitting to standing by pulling a built-in hydraulic lever. Standing posture is stabilized by fixing the trunk, pelvis and knees using front, lateral and back trunk pads, lateral pelvic pads, and kneepads. Bilateral handles located in front of the trunk are linked to the footplates, thus allowing one leg to move forward while the other moves back by pushing and pulling the handles alternately. In the present study, an investigator (HO) manually pulled the hydraulic lever and moved the handles.

The subjects underwent PWM and QS trials (Figure 1). These two trials were performed on separate days within three weeks but in the same time slot. The order of the two trials was randomized. As for the reproducibility of the HR variability indices on the two separate days, intraclass correlation coefficients have been reported to be approximately 0.5 to 0.6 in 456 male students aged 20 to 29 years [9].

In the PWM trial, subjects spent 5 minutes in a sitting position and this was followed by a 5-minute QS period to determine baseline levels in sitting and standing states with a 1-minute transition period for postural change from sitting to standing. The subjects then underwent 25-minute rhythmic PWM at 1 Hz. The hip joint range of motion was set at 30°. The protocol of the QS trial was the same except that the subjects underwent 25-minute QS instead of 25-minute PWM. The subjects were occasionally instructed to relax their body during the experiment.

During PWM, the investigator always checked angle data displayed on an oscilloscope to maintain the predetermined pattern (that is, hip joint range of motion and swing frequency). The angle was measured by an electrogoniometer (Goniometer system, Biometrics Ltd, Ladysmith, VA, USA) placed at the junction of the handle bar and the bar linked to a kneepad. The investigator (HO) conducted a sufficient number of practice sessions before the main tests so that he could adjust the leg motion to the predetermined pattern by monitoring the angle data displayed on the oscilloscope.

### Measurements

Systolic and diastolic arterial blood pressures (SAP and DAP, respectively) were determined noninvasively using an electro-sphygmomanometer (Tango^+^, Sun Tech Medical, Inc. Morrisville, NC, USA). A pneumatic cuff was fixed to the left upper arm, and Korotkov sound was detected by a lavalier microphone fixed on the left brachial artery. The sampling interval was set at 1 minute because it took about 30 sec to terminate one data sampling. Mean arterial blood pressure (MAP) was calculated as DAP plus one third of pulse pressure.

An electrocardiogram was obtained at a sampling rate of 1000 Hz using a bioamplifier (Dual BIO Amp, AD Instruments Pty Ltd, Bella Vista, NSW, Australia).

Continuous surface electromyograms of the rectus femoris muscle, biceps femoris muscle, medial gastrocnemius muscle and tibialis anterior muscle of the left leg were recorded at a sampling rate of 1000 Hz in order to check voluntary or involuntary muscle contraction using a bioamplifier (AB-621 G, Nihon Kohden, Shinjuku-ku, Tokyo, Japan). If marked electromyographic activity was observed, the subjects were asked to relax their leg.

### Data analysis

Beat-to-beat R-R intervals in the 5-minute sitting baseline period, 5-minute standing baseline period, and initial, middle and last 5-minute periods of 25-minute PWM or QS (2 to 6 minutes, 11 to 15 minutes and 21 to 25 minutes, respectively) were derived and were used for calculation of HR. R-R interval with an abnormal value due to ectopy was derived from visual inspection and was suitably corrected. The number of abnormal values was less than 1% of total R-R intervals in each of the 5-minute segments. For each segment, the time series of R-R intervals was cubic-spline interpolated, and then resampled at 2 Hz to create a uniform time series for spectral analysis. Resampled 600-point data were detrended and padded with trailing zeros to 1024-point data. After Hanning windowing, a fast-fourier transform spectrum was calculated. The areas of the low-frequency band (LF) (0.04 to 0.15 Hz) and high-frequency band (HF) (0.15 to 0.4 Hz) were determined, and total power was defined as the sum of LF and HF. Normalized HF (HF_norm_) was calculated as the ratio of HF to total power. HF_norm_ was used as a measure of parasympathetic activity [4]. The ratio of LF to HF (LF/HF) was also calculated as an autonomic balance index. A low LF/HF ratio indicates a dominant modulation of the parasympathetic nervous system, while a high LF/HF ratio indicates a dominant modulation of the sympathetic nervous system [10].

For each of the cardiocirculatory values excluding HF_norm_ and LF/HF, the averages for the 5-minute sitting baseline period, 5-minute standing baseline period, and initial, middle and last 5-minute periods of 25-minute PWM or QS (2 to 6 minutes, 11 to 15 minutes and 21 to 25 minutes, respectively) were calculated and used for statistical analysis. DP was calculated as the product of HR and SAP [5]. For calculation of DP, the 5-minute average of SAP and 5-minute average of HR were used.

Data on cardiocirculatory valuables were analyzed through a two-way repeated measures analysis of variance with type of experimental trial (PWM vs. QS trial) and condition within an experimental trial (sitting vs. standing vs. PWM or QS in the initial period vs. PWM or QS in the middle period vs. PWM or QS in the last period). If an interaction was found, the Bonferroni correction for multiple comparisons was used to determine the difference in the values between two time periods within an experimental protocol. Differences in the values at each time period between the two trials were assessed using the paired *t*-test. A value of *P* <0.05 was regarded as statistically significant. All data are presented as means ± SD.

## Results

In the present study, two subjects complained of feeling discomfort during the 25-minute QS. These subjects were rapidly returned to the sitting position (in the 11 to 12th minute in one subject and in the 17 to 18th minute in the other subject). In one subject, HR at 1 minute before interruption was 111 bpm, falling to 71 bpm just before interruption. In that subject, SAP, DAP and MAP just after interruption were 59, 48 and 52 mmHg, respectively, despite the fact that SAP, DAP and MAP during the sitting baseline period were 108, 66 and 80 mmHg, respectively. In addition, that subject lost consciousness just after interruption. In another subject who dropped out of the 25-minute QS, HR at 1 minute before interruption was 83 bpm, falling to 73 bpm just before interruption. In that subject, values of blood pressure could not be obtained due to rapid discontinuation of data collection. The consciousness was retained. On the other hand, the two subjects could complete the 25-minute PWM.

**Figure 2:**
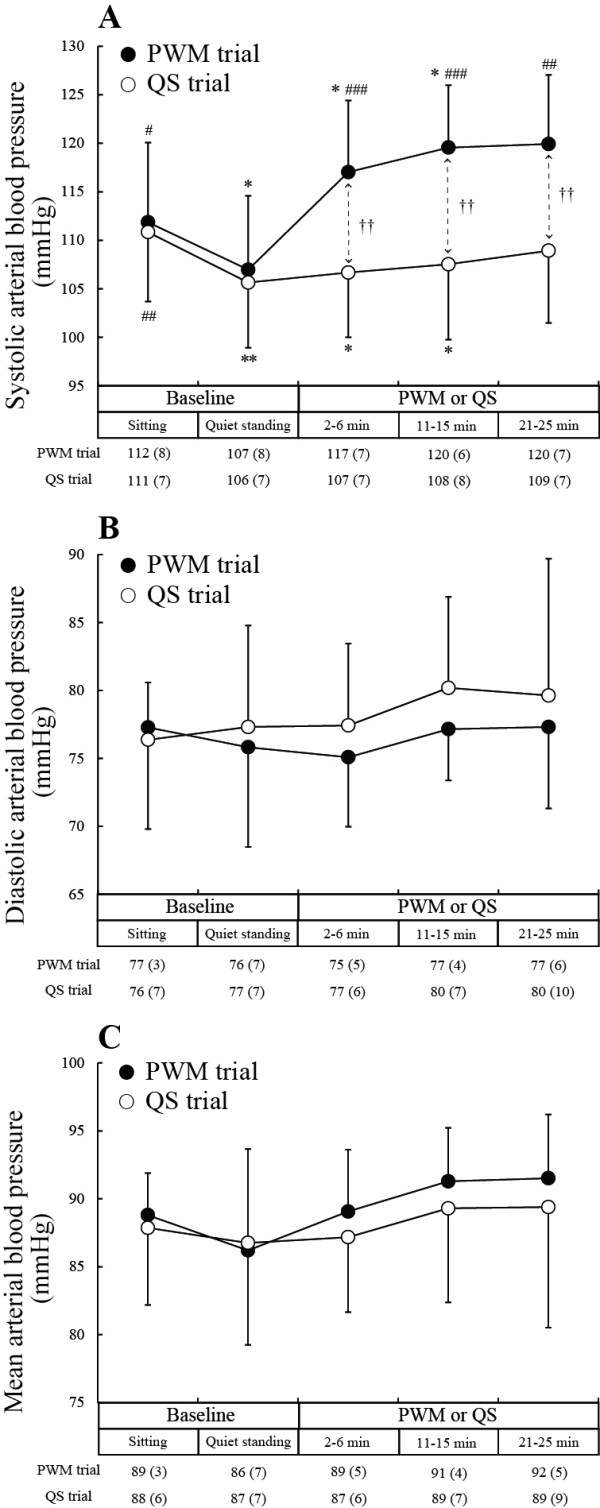
**Changes in systolic, diastolic and mean arterial blood pressure.** PWM: passive walking-like leg movement. QS: quiet standing. *P < 0.05, **P < 0.01, ***P < 0.001 compared to the sitting baseline level; ^#^*P* < 0.05, ^##^*P* < 0.01, ^###^*P* <0.001 compared to the quiet standing baseline level; †*P* < 0.05, †† *P* < 0.01, †††*P* < 0.001 for comparison between values in the PWM and QS trials.

Since data collection in the two subjects was discontinued, statistical analysis of responses was made using data obtained in the remaining eleven subjects. Significant interactions between type of experimental trial and condition within an experimental trial were observed for SAP, DP, HR, LF/HF and HF_norm_. There was no significant change in DAP or MAP (Figure 2B and C).

Figure 2A shows changes in SAP. The SAPs during the standing baseline period were significantly lower than those during the sitting baseline period in both the PWM trial (*P* < 0.05) and QS trial (*P* < 0.01). The magnitude of decrease in SAP was 5 ± 4 mmHg and 5 ± 3 mmHg in the PWM and QS trials, respectively. SAP remained lowered during the initial and middle periods of the 25-minute QS compared to the sitting baseline level (*P* < 0.05), whereas SAP during PWM tended to be higher than that during the sitting baseline period due to an average increase in SAP of 12 ± 7 mmHg from the level during the standing baseline period. SAPs at each of the periods during the 25-minute PWM were significantly higher than those at each of the corresponding periods during the 25-minute QS (*P* < 0.01).

**Figure 3:**
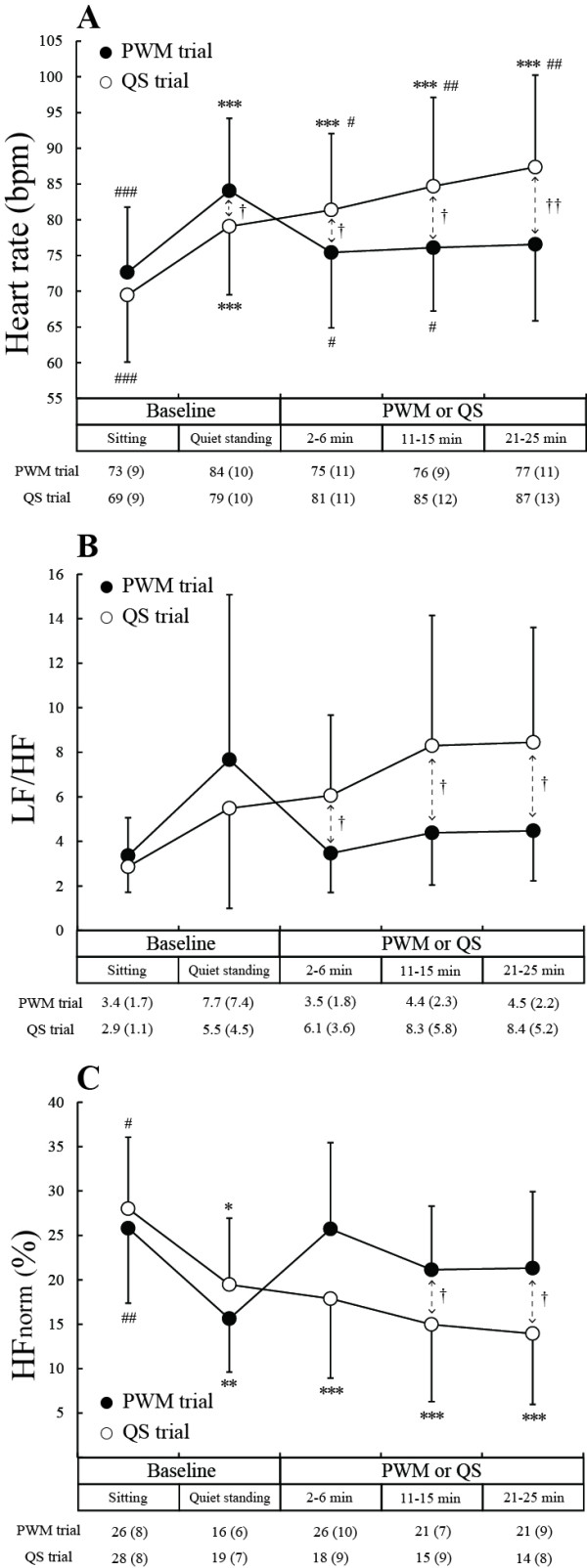
**Changes in heart rate, the ratio of low-frequency component to high-frequency component of the heart rate variability spectrum (LF/HF), and high-frequency component of the heart rate variability spectrum normalized by total power (HF**_**norm**_**).** PWM: passive walking-like leg movement. QS: quiet standing. *P < 0.05, **P < 0.01, ***P < 0.001 compared to the sitting baseline level; ^#^*P* < 0.05, ^##^*P* < 0.01, ^###^*P* <0.001 compared to the quiet standing baseline level; †*P* < 0.05, †† *P* < 0.01, †††*P* < 0.001 for comparison between values in the PWM and QS trials.

Figure 3A shows changes in HR. The HR during the standing baseline period was significantly higher than during the sitting baseline period in both the PWM and QS trials (*P* < 0.001). The magnitude of increase in HR was 11 ± 3 bpm and 10 ± 4 bpm in the PWM and QS trials, respectively. HR remained above the sitting baseline level throughout the 25-minute QS (*P* < 0.001), whereas HR during the 25-minute PWM did not differ significantly from that during the sitting baseline period due to a significant average decrease in HR (*P* < 0.05) of 8 ± 7 bpm from the level during the standing baseline period. HR at each of the periods during 25-minute PWM was significantly lower than at each of the corresponding periods during 25-minute QS regardless of the fact that HR during the standing baseline period was significantly higher in the PWM than in the QS trial.

Figure 3B and C shows changes in LF/HF and HF_norm_. The patterns of change in LF/HF were similar to the patterns of changes in HR, while the patterns of change in HF_norm_ were almost the mirror images of the patterns of change in HR. HF_norm_ during the standing baseline period was significantly lower than during the sitting baseline period in both the PWM trial (*P* < 0.01) and QS trial (*P* < 0.05). HF_norm_ remained below the sitting baseline level throughout the 25-minute QS (*P* < 0.001), whereas HF_norm_ during PWM showed no significant difference from HF_norm_ during the sitting baseline period. HF_norm_ during the middle and last periods of 25-minute PWM was significantly higher than during the corresponding periods of 25-minute QS (*P* < 0.05). On the other hand, LF/HF at each of the periods during 25-minute PWM was significantly lower than at each of the corresponding periods of 25-minute QS (*P* < 0.05).

Since the power spectral density of HR variability has a peak at the center of respiratory frequency in the HF region, we visually identified a peak in the HF region of the power spectrum to estimate respiratory frequency. A distinct peak of all five periods in both the PWM and QS trials was observed in nine of the eleven subjects. The estimated respiratory frequencies during the sitting and standing baseline periods and during the first, middle and last periods in the PWM trial were 18.2 ± 3.2, 18.3 ± 3.9, 19.4 ± 3.0, 20.5 ± 2.8 and 20.4 ± 3.1 times/minute, respectively, while those in the QS trial were 18.3 ± 3.5, 17.7 ± 4.3, 17.2 ± 4.3, 18.0 ± 4.3 and 19.0 ± 4.6 times/minute, respectively. There was no significant change in estimated respiratory frequency in the PWM or QS trials. The patterns of change in HF_norm_ in the PWM and QS trials in the nine subjects were the same as those shown in Figure 3C: there was no significant difference in HF_norm_ during the sitting baseline period and during the 25-minute PWM in the PWM trial, and the decrease in HF_norm_ during the standing baseline period was sustained during the subsequent 25-minute QS in the QS trial.

**Figure 4:**
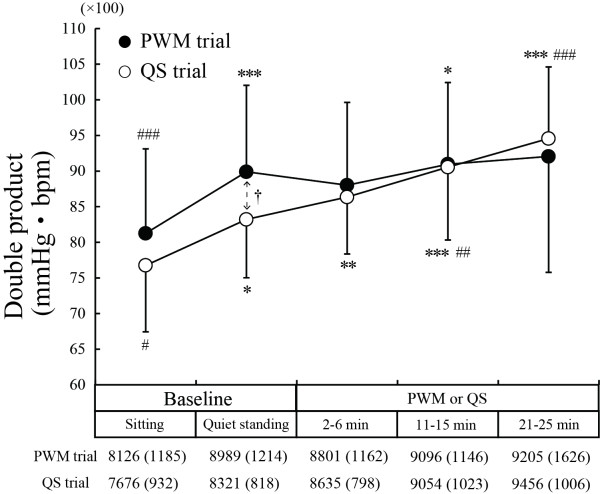
**Change in double product.** PWM: passive walking-like leg movement. QS: quiet standing. *P < 0.05, **P < 0.01, ***P < 0.001 compared to the sitting baseline level; ^#^*P* < 0.05, ^##^*P* < 0.01, ^###^*P* <0.001 compared to the quiet standing baseline level; †*P* < 0.05, †† *P* < 0.01, †††*P* < 0.001 for comparison between values in the PWM and QS trials.

Figure 4 shows changes in DP. The DP during the standing baseline period was significantly higher than during the sitting baseline period in both the PWM trial (*P* < 0.001) and QS trial (*P* < 0.05) due to an increase in HR (Figure 3A). In the QS trial, DP during the middle and last periods of 25-minute QS was significantly higher than DP during the standing baseline period (middle period, *P* < 0.01; last period, *P* < 0.001) due to an increase in HR. In the PWM trial, on the other hand, DP remained almost constant during the 25-minute PWM, thus showing no significant difference from the level during the standing baseline period.

## Discussion

### Mechanisms responsible for cardiocirculatory responses to PWM

An increase in HR and a decrease in HF_norm_ with postural change from sitting to standing observed in the present study is in accordance with responses observed in previous studies [10,11]. The lower HF power during standing reflects withdrawal of vagal activity [10]. The reduction in vagal activity must have caused an increase in HR during QS in the present study.

In accordance with the results of a previous study [1], we observed an increase in SAP and a decrease in HR during PWM compared to the levels during QS. We also found that the decrease in HF_norm_ with postural change from sitting to standing was suppressed by PWM. Similar observations were reported by Niizeki *et al*. [11]. They determined the muscle pump effect of the leg during QS on cardiac autonomic activity in healthy subjects. The muscle pump was simulated by rhythmic lower-leg cuff inflation. Compared to the sham condition (no cuff pressure), HR decreased and stroke volume increased in the condition with cuff inflation above 80 mmHg. The decrease in HR was accompanied by an increase in the high-frequency component of HR variability. Niizeki *et al*. [11] proposed the following mechanisms for the circulatory responses. The application of cuff inflation increases venous return by the mechanical pumping effect of inflation. Increased venous return increases afferent input from cardiopulmonary baroreceptors, which would inhibit efferent sympathetic nerve activity, resulting in a shift of the sympathovagal balance toward a parasympathetic activation.

In the present study, venous return and stroke volume were not measured, but lower-leg blood volume measured by near-infrared spectroscopy was found to be decreased during PWM [12], suggesting an increase in venous return from the leg. Considering the similarity of cardiocirculatory responses observed in the study by Niizeki *et al*. [11] and in our study, it is likely that a mechanism similar to that proposed by Niizeki *et al*. [11] was operative during PWM in the present study.

In the present study, LF/HF during 25-minute PWM was lower than that during 25-minute QS. This reflects a dominant modulation of the parasympathetic nervous system relative to the sympathetic nervous system during PWM.

Although our findings were in accordance with the findings by Niizeki *et al*. [11], pressor response, which was absent during rhythmic cuff inflation in the study by Niizeki *et al*. [11], occurred during PWM in the present study. It is thought that the pressor response to PWM is caused not only by the muscle pump effect but also a brainstem-mediated neural reflex mechanism, the afferent signals being originated from the passively moved leg [1]. The latter mechanism may have a major effect on the pressor response to PWM.

### Implications

Two subjects dropped out of the 25-minute QS. Those subjects showed an abrupt decrease in HR just before interruption. In addition, one of those subjects clearly showed an extreme reduction in blood pressure and lost consciousness just after the interruption. These responses should be vasovagal responses [6]. However, since the two subjects could complete the 25-minute PWM, PWM can serve as a maneuver for prevention of vasovagal response. This idea is also supported by cardiocirculatory responses to PWM for the following reasons. First, PWM can induce pressor response, thus counteracting hypotension during vasovagal response. Second, the most commonly used model for triggering vasovagal response is the Bezold-Jarisch reflex. A literature review by Fenton *et al*. [2] summarized the mechanism underlying the Bezold-Jarisch reflex as follows. Upright posture causes a pooling of blood in the lower extremities, decreasing blood volume in the ventricle and at the level of the aortic arch and carotid sinus. This results in decreased afferent neural activity from baroreceptors, producing increased sympathetic tone to the vasculature and the heart, with subsequent vasoconstriction and increased inotropy and HR. The Bezold-Jarisch reflex is initiated by a decrease in ventricular volume and an increase in ventricular inotropy. This activates sensory receptors that respond to wall tension located in the inferoposterior portion of the left ventricle, paradoxically increasing neural traffic to the central nervous system through afferents in the vagus nerve. Sympathetic output to the vasculature and the heart decreases, and parasympathetic activity increases. On the other hand, PWM can induce physiological responses against the Bezold-Jarisch reflex, that is, reduction of blood volume in the leg [12] and maintenance of parasympathetic activity at the level during sitting (Figure 3C). The former response would cause a decrease in venous pooling with resulting increase in ventricular volume, and the latter response would enable ventricular inotropy to be kept at the level during sitting.

DP is a predictor of myocardial oxygen consumption in normal young men during upright exercise under a wide variety of circumstances [5]. In the present study, DP remained almost constant during the 25-minute PWM in contrast to the fact that DP increased gradually during the 25-minute QS, suggesting that PWM enables cardiac activity to be kept at a constant level. This stability may also be effective for prevention of vasovagal response.

In the review by van Dijk [13], orthostatic syncope is discussed from the viewpoint of physiological anthropology: orthostatic syncope has not been described in apes; an orthostatic fainting tendency in man may be due to the large amounts of venous pooling in the legs regardless of the fact that the large proportion of cardiac output (20%) is directed to the brain; the effect of this anatomical characteristic has not been corrected for by altered physiology because human anatomy has a history of only 100,000 to 200,000 years. It is conceivable that human walking-like leg movement was developed to compensate for the lack of physiological adaptation to upright posture.

### Limitations

A limitation of the present study is that HR during the standing baseline period was higher in the PWM trial than in the QS trial, resulting in higher DP in the PWM trial, although the first 10-minute period, including a 5-minute sitting baseline period and subsequent 5-minute standing baseline period, was the same condition in the PWM and QS trials. When HR and DP during the standing baseline period were expressed as percentages of those during the sitting baseline period, there was no significant difference in HR or DP between the two trials (HR 116 ± 5% vs. 114 ± 7% in the PWM trial and QS trial, respectively; DP 111 ± 6% vs. 109 ± 7% in the PWM trial and QS trial, respectively). Thus, the higher HR and DP during the standing baseline period would have been caused by larger baseline HR level on the day of the PWM trial possibly due to day-to-day variation. Nevertheless, we could clearly show different patterns of changes in HR and DP in the two trials, that is, HR and DP increased gradually with time in the QS trial, whereas such increases in HR and DP were suppressed in the PWM trial.

Another limitation of this study is the absence of respiratory adjustment during the experimental interventions, thus making changes in the HF component of HR variability difficult to interpret. Hayano and Yasuma [14] proposed a model of central regulation of cardiac vagal outflow. This model is composed of a tonic control system and a phasic control system. The former system regulates the mean level of cardiac vagal outflow (cardiac vagal tone). Bradycardia, as shown during PWM, is derived from this system. The latter system regulates the amplitude of respiratory modulation of cardiac vagal outflow. Brown *et al*. [15] have shown that an increase in tidal volume enhances the power of HR variability at respiratory rate, whereas an increase in respiratory frequency diminishes the power of HR variability at respiratory rate. Since we did not instruct subjects to maintain constant breathing frequency and/or breathing depth, suppression of the decrease in HF_norm_ by PWM might be influenced not only by vagal tone but also by respiratory modulation of cardiac vagal outflow through increased tidal volume and/or decreased respiratory frequency. In the present study, however, suppression of the decrease in HF_norm_ during PWM was not accompanied by a significant change in estimated respiratory frequency. In addition, we previously found no significant change in respiratory frequency or tidal volume during 12-minute PWM compared to values during preceding 6-minute QS in twelve healthy subjects with a mean age of 33 ± 8 years (unpublished data from a previously published study [1]), although those subjects were older than in the present study. Thus, we believe that suppression of the decrease in HF_norm_ during PWM is largely due to an increase in vagal tone.

## Conclusions

A reduction in SAP and an increase in HR due to postural change from sitting to standing are suppressed by PWM in healthy young men. A reduction in HR by PWM is due to parasympathetic activation. Myocardial oxygen consumption remains almost constant during PWM, although myocardial oxygen consumption during QS is gradually increased with time. It is thought that PWM suppresses cardiocirculatory responses to orthostatic stress and thus enables young people to keep the standing posture for a longer time with lower risk of vasovagal response.

## Abbreviations

DAP: diastolic arterial blood pressure; DP: double product; HF: area of the high-frequency band (0.15-0.4 Hz) of a fast-fourier transform spectrum; HF_norm_: normalized HF (HF/(LF + HF)); HR: heart rate; LF: area of the low-frequency band (0.04-0.15 Hz) of a fast-fourier transform spectrum; LF/HF: Ratio of LF to HF; MAP: mean arterial blood pressure; PWM: passive walking-like leg movement in the standing posture; QS: quiet standing; SAP: systolic arterial blood pressure; SD: standard deviation.

## Competing interests

The authors declare that they have no competing interests.

## Authors' contributions

HO contributed to all of the works, including conception, design, acquisition of data, execution of the experiment (Imposition of passive leg movement), analysis and interpretation of data, and writing the paper. IF and KY contributed to design and execution of the experiment (Imposition of passive leg movement and management of risks of orthostatic intolerance). TK contributed to execution of the experiment (Management of risks of orthostatic intolerance) and helped to draft the manuscript. All authors read and approved the final manuscript.
